# High-Yield Production of Aqueous Graphene for Electrohydrodynamic Drop-on-Demand Printing of Biocompatible Conductive Patterns

**DOI:** 10.3390/bios10010006

**Published:** 2020-01-17

**Authors:** Amir Ehsan Niaraki Asli, Jingshuai Guo, Pei Lun Lai, Reza Montazami, Nicole N. Hashemi

**Affiliations:** 1 Department of Mechanical Engineering, Iowa State University, Ames, IA 50011, USA; niaraki@iastate.edu (A.E.N.A.); jguo1@iastate.edu (J.G.); issaclun@iastate.edu (P.L.L.); reza@iastate.edu (R.M.); 2 Department of Biomedical Sciences, Iowa State University, Ames, IA 50011, USA

**Keywords:** graphene, inkjet printing, conductive ink, flexible electronics, neuronal sensing

## Abstract

Presented here is a scalable and aqueous phase exfoliation of graphite to high yield and quality of few layer graphene (FLG) using Bovine Serum Albomine (BSA) and wet ball milling. The produced graphene ink is tailored for printable and flexible electronics, having shown promising results in terms of electrical conductivity and temporal stability. Shear force generated by steel balls which resulted in 2–3 layer defect-free graphene platelets with an average size of hundreds of nm, and with a concentration of about 5.1 mg/mL characterized by Raman spectroscopy, atomic force microscopy (AFM), transmittance electron microscopy (TEM) and UV-vis spectroscopy. Further, a conductive ink was prepared and printed on flexible substrate (Polyimide) with controlled resolution. Scanning electron microscopy (SEM) and Profilometry revealed the effect of thermal annealing on the prints to concede consistent morphological characteristics. The resulted sheet resistance was measured to be $R_{s}\;=\;36.75\;\Omega /\mathrm{sqr}$ for prints as long as 100 mm. Printable inks were produced in volumes ranging from 20 mL to 1 L, with potential to facilitate large scale production of graphene for applications in biosensors, as well as flexible and printable electronics.

## 1. Introduction

Printable electronics have received increasing attention due to their broad applications, such as roll-to-roll (R2R) printed solar cells [1], micro electrode array (MEA) [2], biomedical/chemical sensors [3] and the manufacturing of various flexible electronics [4,5,6]. As the printable inks are core components in this field of research, several studies have been conducted on increasing the conductivity and printability of these materials. Metal nanoparticles [7] and nanowires [8], conductive polymers [9] and Indium Tin Oxide (ITO) are the most common conductive inks. However, the natural brittleness of these materials (e.g., ITO) hinders their applicability to the flexible substrate [10]. In biological sensing applications, utilizing these materials alters the cell shape, organization and function of the cell culture, therefore establishing a reliable communication remains a challenge due to this mechanical mismatch [2].

Graphene-based inks have shown promise in fulfilling the aforementioned needs [11,12]. Graphene is a one-atom-thick, two-dimensional, honey-combed arrangement of hybridized carbon atoms, the large scale production of which has remained a challenge since 2004 [13,14,15]. Researchers have synthesized graphene by the oxidation of graphite through the modified Hummer’s method, chemical vapor deposition (CVD) from hydrocarbon gas and liquid-phase exfoliation [14,15,16,17,18]. 

At this time, it is evident that the Hummer’s method causes single carbon atom defects and nano-sized holes, due to over-oxidization of the carbon framework [19]. As graphene oxide is insulating, oxidation is commonly unwanted in electronic applications. Although there have been notable improvements on its processability, the electronic characteristics of these graphene-based inks are not equal to those of pristine graphene, even after chemical reduction [20]. On the other hand, large, continuous graphene films can be created by CVD, however, this method has shown limited success due to the presence of numerous surface voids and defects [21]. Additionally, one of the issues with transferring CVD graphene into the desired substrate is the unwanted residues of the etching agents [22,23,24].

Direct Liquid Phase Exfoliation (LPE) of graphite into graphene has been broadly reported to be desirable for inkjet printing, particularly so for electrophysiology and cell-based studies [25,26,27,28]. To this date, there are several reported LPE methods, all of which vary in resultant quality and yield [21,28,29]. Popular graphene solvents such as dimethylformamide (DMF) and N-methylpyrrolidone (NMP) [30] often result in low graphene concentration in the conductive ink ($\sim 0.01\;{\mathrm{mg}\;\mathrm{mL}}^{-1}$) [28], and possess very low viscosity (<2 cP), placing them far from practical applications [31]. Furthermore, DMF and NMP are toxic, and in consequence, detrimental to cell cultures [32]. Since most biological media and cell cultures are aqueous, the stable and processable dispersion of aqueous graphene is essential to facilitate the use of this material in biomedical applications [13,16,17,18,23,24,25,26,27,33]. Fortunately, in numerous electroceutical studies, it is shown that excitable cells grow compatibly with the presence of graphene in the extra-cellular environment [34,35]. However, graphene platelets do not naturally remain suspended in water and aggregate due to the interplanar Van der Waals interactions.

In order to overcome this issue, Paton et al. successfully exhibited the usage of an edible protein, bovine serum albumin (BSA), as a stabilizing agent in the LPE process [36]. Kumar et al. have shown that BSA among other proteins, facilitates graphite exfoliation with the highest throughput [37]. Khademhosseini et al. produced graphene using BSA and sonication to exfoliate graphite for biomedical purposes [38]. Nevertheless, it has become evident that continuous sonication is ineffective for providing a higher graphene yield, and excessive sonication can result in damage to graphene [28,39].

It has been long known that other mechanical activation techniques, such as ball milling, can be considered as a promising process for modifying carbon nanostructures [40]. To the knowledge of the authors, thus far ball milling has been used to exfoliate graphite in solid condition with melamine in two separate studies, resulting in a maximum concentration of $0.13\;{\mathrm{mg}\;\mathrm{mL}}^{-1}$ [41], and $0.37{\;\mathrm{mg}\;\mathrm{mL}}^{-1}$ [42], in DMF. Wet ball milling was utilized to produce FLG in NMP [43]. Edge-carboxylated graphene nanosheets can be produced via ball milling in dry ice, which results in a very high conductivity, but with the concentration of $0.06{\;\mathrm{mg}\;\mathrm{mL}}^{-1}$ [44]. In consequence, attaining higher concentrations of graphene as a conductive ink, while suspending graphene sheets in water remains a desired task in hand.

In the course of printing, a high concentration of graphene in solution serves a threefold purpose. Firstly, the more concentrated the graphene solution, the more viscose the ink, which in practice should reach to approximately 10 cP [32]. Adversely, the low concentration of graphene demands several tens of print passes for obtaining functional films. Finally, concentration of graphene is correlated with the conductivity of the ink, which serves as a control factor in the electrostatic field induced by the drop-on-demand systems [14].

Herein, we initially propose the combination of BSA as an exfoliating/stabling agent, with the sheer force of continuous low speed wet ball milling for achieving scalable and stable water-dispersed graphene nanosheets with high yield. Next, through utilizing an electrostatic field, the inkjet printing of binder-free graphene solution on flexible substrate is demonstrated. To end with, the resultant conductivity of the printed circuit is characterized, and its stability after submergence in water is exhibited. In another study by our group, the biofunctionalization of the biosensors produced via this process is exhibited [45].

It is shown that rat neuronal cells can be cultured in-vitro on graphene biosensors, and these sensors can enable the sensing of electrical signals on a cell membrane. This study aims to provide a practical guideline for the production of highly concentrated graphene and the patterning of circuits for use in biosensing and other applications in flexible electronics.

## 2. Materials and Methods

**Figure 1:**
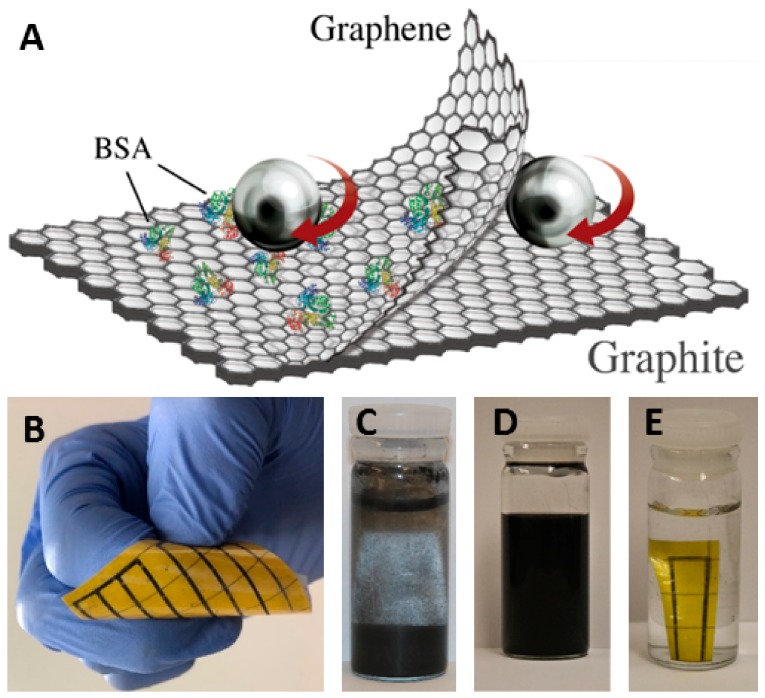
Shear exfoliation of graphene. (**A**) Schematics of the shear exfoliation of graphite using steel balls (**B**) Printed graphene ink on flexible substrate (**C**) Graphite powder in water before exfoliation (**D**) Graphene ink with concentration of 5.1 ${\mathrm{mg}\;\mathrm{mL}}^{-1}$ after remaining still for one month. (**E**) Stability of graphene patterns after 7 days of submergence in water.

Graphite crystallites (≈20 μm) were exfoliated and broken down to FLG platelets via the shear tension created by the abrasion of steel balls with the diameter of $11/32^{\prime \prime }$ (Figure 1A). Since turbulent energy dissipation is not necessary for exfoliation [36], the rotational speed was fixed as low as 300 rpm for 90 h in all trials to prevent undesired temperature spikes. The proportion of Graphite (${\left[Graphite\right]}_{0}=\;20{\;\mathrm{mg}\;\mathrm{mL}}^{-1})$, BSA (${\left[BSA\right]}_{0}=\;2{\;\mathrm{mg}\;\mathrm{mL}}^{-1}$) and $r_{bs}=\;500\;\pm \;10{\;m}^{2}/m^{3}$ were constant for all of the results reported in this study, where $r_{bs}$ is the ratio of the overall surface area of the balls with respect to the solution volume.

BSA is an inexpensive protein and most commonly found as a waste product in the meat industry [46,47], which plays a key role in preventing the aggregation of exfoliated platelets. While the large hydrophobic surface area of graphene nanosheet bonds with the hydrophobic segment of BSA, the hydrophilic fragment of BSA interacts with water molecules. It is important to note that, through interactions with BSA, the structural and intrinsic properties of graphene nanosheets alters minimally, as it is believed that BSA enables the stabilization of graphene through non-covalent bonding [48].

The ball milled solution was allowed to rest for ~48 h, as it is evident that not all of the graphite particles can be exfoliated to the desired FLG. Therefore, the thicker graphene sheets, having failed to maintain their bond with BSA, aggregated with unbroken graphite particles and settled prior to the separation of graphene solution from its sediment. The solution was further centrifuged at 1500 rpm for 45 min, and 85% of the volume from the top was pipetted off. The purpose of this centrifugation is both to remove the remaining graphite particles from the solution and to ensure no Fe impurity from the steel balls can contaminate the samples.

Finally, the concentration of the resultant graphene solution was investigated by UV-vis spectroscopy in different batch sizes to demonstrate the scalability of the method.

The quality of initial graphite crystallites plays a significant role in the quality of the resultant graphene solution [37], and the further studies on the influence of graphite on the produced graphene is left to be addressed elsewhere. The quality of the produced graphene was examined by UV-Vis spectroscopy, Raman spectroscopy, TEM and AFM, as given below.

Absorption spectra were recorded on a PerkinElmer UV-Vis spectrophotometer (Lambda 750) at λ = 660 nm in room temperature. The value of the absorbance coefficient α was obtained by measuring the absorption of the dispersion per length A/L, at various controlled dilutions: $0.05,\;0.08,\;0.11,\;0.14,\;0.17\;\mathrm{and}\;0.20{\;\mathrm{mg}\;\mathrm{mL}}^{-1}$. The mass of the remaining film after the evaporation of water in controlled concentrations was measured for 5 mL samples. The resultant absorption coefficient of $\alpha \;=\;3525{\;\mathrm{mL}\;\mathrm{mg}}^{-1}m^{-1}$ was implemented in the Beer–Lambert law to give resultant concentration measurements (C = αA/L) [36].

Raman spectra of the thin films on alumina membranes were acquired using a BWTEK Voyage confocal Raman system (B&W Tek, Newark, DE, USA), with a CW laser (Excelsior-532-150-CDRH Spectra-Physics) as the energy source operating at the wavelength of 532 nm. Graphene ink drops were dried on $\mathrm{Si}/{\mathrm{SiO}}_{2}$ glass chips with diameter ≈10 mm. There were little variations in the spectra acquired in different laser spots, thus an average of five different points on each sample was reported here.

TEM images were recorded using a JEOL JSM2100 STEM (Japan Electron Optics Laboratories, Mitaka, Tokyo, Japan) at 200 kV accelerating voltage. Graphene samples were diluted to $20{\;\mu g\;\mathrm{mL}}^{-1}$, and were drop casted on a Cu-grid. The AFM images were captured on a Bruker dimension Icon AFM in contact mode and were analyzed by NanoScope Analysis software, version 1.50. Optical microscopy images were recorded by using a Zeiss Axio Observer Z1 Inverted Microscope (Zeiss, Jena, Germany). The waviness and roughness of graphene prints were monitored via a non-contact optical Profilometer, Zygo 7100. We reduced the effect of local roughness to find the cross-sectional profile of prints by calculating average of the heights in circles with radii of 1 μm, and their centers are located in the line demonstrated in Figure S4. SEM Images of printed graphene patterns on PI were captured via a JEOL FESM 6335 using 2–5 kV accelerating voltage. The resistance measurements were conducted by a VersaSTAT 4 Potentiostat Galvanostat.

The physical properties of graphene ink itself are critically influential in controlling the resolution and consistency of the printed patterns. Hence, two nondimensional properties: Reynolds number and Weber number (Equations (1) and (2)) were utilized to govern the printability of the ink [49]. Consequently, the flow rate and the intensity of the electrostatic field applied to the substrate were tuned in the experiments, resulting in average Re=45.1 and We=33.7 for the reported ink and setup. Reynolds and Weber numbers are related by the Ohnesorge number (Oh), which describes the jettability of an ink regardless of the velocity of ink drops [50]. Suggested by Derby et al. for proper jetting to occur, the *Z*-value (inverse of O h) should be between 1 and 10, and the drop impact ($K_{c}$) be below 100 [49].

(1) $$Re\;=\;\frac{v\rho a}{\mu }$$

(2) $$We\;=\;\frac{v^{2}\rho a}{\gamma }$$

(3) $$Z\;=\;\frac{1}{Oh}\;=\;\frac{Re}{\sqrt{We}}\;=\;\frac{{\left(\gamma \rho a\right)}^{\frac{1}{2}}}{\mu };\;1\;<\;Z\;<\;10$$

(4) $$K_{c}\;=\;We^{0.5}Re^{0.25};\;K_{c}\;<\;100$$

where v is the impact velocity, ρ is the ink density, a is the diameter of jetting nozzle, μ is the viscosity of the ink and γ is the surface tension. The conditions for the *Z*-value and the drop impact, given in Equations (3) and (4), were satisfied to obtain a printable ink. These equations confirm that the ink is not too viscous to clog the needle or the junctions, and not too diluted to splash while maintaining high flow rates. 

A high flow rate is essential for achieving superior conductivity outcomes for single printing passes. The measured Re and We for the reported ink and setup was 25.1 and 33.7, respectively. These were measured at room temperature, resulting in a *Z*-value of Z = 4.32 and a drop impact of $K_{c}\;=\;12.99$, which satisfies the suggested conditions.

Polyimide film has a hydrophobic surface which requires surface modification for inkjet printing. PI film was washed with water and acetone before plasma cleaning, then submerged in a solution of PSS in DI water *(*$12\;{\mathrm{mg}\;\mathrm{mL}}^{-1}$) and NaCl ($0.5\;{\mathrm{mol}\;L}^{-1})$ for 20 min followed by submergence in a solution of PEI in DI water ($30\;{\mathrm{mg}\;\mathrm{mL}}^{-1}$) and NaCl ($0.5\;{\mathrm{mol}\;L}^{-1})$ for another 20 min. The substrates were finally thoroughly washed with DI water and dried by pressurized nitrogen. In the course of printing, a 5 mL Syringe filled with graphene ink was fixed in a syringe pump, to inject the ink with the rate of $9\;{\mu L\;s}^{-1}$ for needles with an inner diameter of 300 μm. Kapton PI tape was fixed on an aluminum film, and a 3 kV potential difference was applied between the tip of the needle and the aluminum film to adhere the ink onto the substrate. The position of the tip of the needle was controlled through a Computer Numerical Control (CNC), and all of the printed graphene is the result of one pass of the needle over the substrate for 120 mm with the movement speed of 10 mm/s.

Graphite powder (≈20 μm), BSA, Poly 4-styrenesulfonic acid sodium salt (PSS), Poly ethyleneimine (PEI) $50\%\;\left(w/v\right)$ in $H_{2}O$, graphene dispersion ($\geq 0.2{\;\mathrm{mg}\;\mathrm{mL}}^{-1}$ in DMF) with sheet resistance of 24.2 kΩ/sqr (as made), 7.9 kΩ/sqr  (after 30 min, 200 °C); and graphene dispersion ($1{\;\mathrm{mg}\;\mathrm{mL}}^{-1}$ in DMF) with sheet resistance of 16 kΩ/sqr (as made), 4.1 kΩ/sqr (after 30 min, 200 °C), were purchased from Sigma-Aldrich (St. Louis, MO, USA).

## 3. Results and Discussion

### 3.1. Graphene Characterization

**Figure 2:**
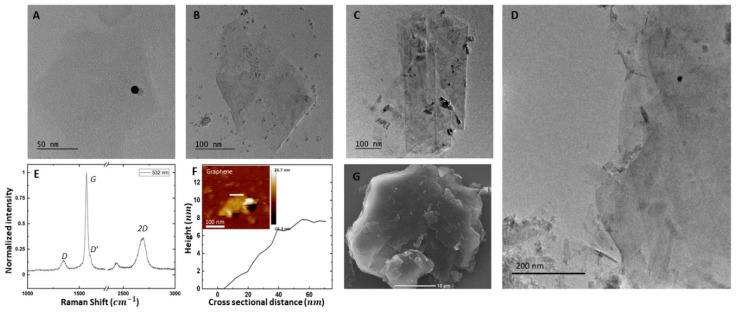
(**A**) Single graphene flake, which is semi-transparent; scale bar: 50 nm. (**B**) Transmission electron microscopy (TEM) image of a single few layer graphene (FLG), which is folded due to its large aspect ratio; scale bar: 100 nm. (**C**) Multiple graphene flakes with crumbled and folded morphology; scale bar: 100 nm. (**D**) TEM imaging demonstrates a distribution of flake sizes that tend to stack up; Scale bar: 200 nm. (**E**) Raman spectra of Graphene sample in 5 different laser spots. (**F**) Atomic force microscopy (AFM) image and corresponding height profile of graphene. (**G**) SEM Image of a Single Graphite particle; scale bar: 10 μm.

Former studies have revealed that the physical properties of graphene-based materials are determined by their structure, the number of hexagonal carbon layers, which also represents thickness and the defects or contaminants present in the material [22]. Raman spectroscopy is the most widely accepted way to gain information on these physical characteristics [51,52,53]. In this study, Raman spectra of the graphene (Figure 2E) indicate that the most noticeable intensities belonged to D ($\approx 1348{\;\mathrm{cm}}^{-1}$), G ($\approx 1569\;{\mathrm{cm}}^{-1}$), D’ ($\approx 1620\;{\mathrm{cm}}^{-1}$) and 2D ($\approx 2695\;{\mathrm{cm}}^{-1}$) bands which typically contain information regarding defects, lateral size and the number of layers of exfoliated graphene platelets. One of the most prominent characteristics for the Raman spectrum of graphene, compared to graphite, is the elimination of the shoulder peak on the 2D band. This is most likely due to growth in the number of aromatic domains [36].

Another significant sign of presence of graphene is the intensity of the D band, which is barely observable in graphite samples, ${\left(I_{D}/I_{G}\right)}_{Graphtie}\;=\;0.02$, and markedly increased to ${\left(I_{D}/I_{G}\right)}_{Graphene}\;=\;0.16$ [38]. In addition, although the G band remained unchanged, there appeared the D’ band shoulder peak. D and D’ bands are created due to the shear force produced by steel balls, which effectively break the graphite crystals into FLG platelets. It was found by Eckmann et al. that the ratio of the intensity values of the D band ($I_{D}$) and the $D^{\prime }$ band ($I_{D}^{\prime }$) can quantitatively denote disorders, vacancy defects and possible oxidization [52], which degrades the electrical characteristics of FLG. By their very nature, graphene nanoplatelets contain edges which act as defects. The type of these defects must be identified, and it should be determined that whether the presence of D band is due to basal plane defects or nano sheet edges, before the graphene materials are to be used. It is known that the values of $\frac{I_{D}}{I_{D^{\prime }}}>3.5,7,13,\;$ respectively characterize boundary defects, vacancy basal plane pint defects and $sp^{3}$ defects [36]. The ratio of these peaks was measured to be $\frac{I_{D}}{I_{D}^{\prime }}<$ 3.5 in all batch volumes, which can be interpreted as no observed $sp^{3}$ defects (oxidization) or vacancy defects during the exfoliation process. Changes of $I_{D}/I_{D}^{\prime }$ in different batch volumes is given in Figure S1.

In order to further confirm the presence of defect-free graphene and the degree of exfoliation, transmission electron microscopy (TEM) images were collected using dried, deposited graphene (20 μg mL^−1^) on a Cu-grid (Figure 2A–D). No basal defects, cracks or holes were observed in graphene nanosheets, supporting the Raman tests. In many instances, the sheets are found to exhibit a folded or crumbled morphology. This could explain the discrepancies for average lateral sizes measured by TEM when compared to Raman results (Figure 2E). Overall, TEM showed that most graphene sheets had a maximum lateral dimension on the scale of hundreds of nm and supported the successful exfoliation of graphite to defect-free FLG.

The number of layers in FLG platelets can be quantitatively estimated based on the shape of the 2D band of both shear exfoliated graphene and source graphite powder given in Equation (5) [36]. *M* is a metric proposed by Paton et al. [36] (Equation (6)), where $\omega_{p}$ is the intensity of the 2D peak and $\omega_{s}=\omega_{p}\;-\;30\;{\mathrm{cm}}^{-1}$ is the intensity of the left hand-side shoulder peak. In various batch sizes, $N_{G}$ was resulted to be 2.9 ± 0.3. It is worth noting that the calculated $N_{G}$ is highly sensitive to parent graphite and can vary in other experiments.

(5) $$\langle N_{G}\rangle \;=\;10^{0.84M+0.45M^{2}}$$

(6) $$M\;=\;\frac{I_{G^{\prime }ene}\left(\omega \;=\;\omega_{p,G^{\prime }ite}\right)/I_{G^{\prime }ene}\left(\omega \;=\;\omega_{s,G^{\prime }ite}\right)}{I_{G^{\prime }ite}\left(\omega \;=\;\omega_{p,G^{\prime }ite}\right)/I_{G^{\prime }ite}\left(\omega \;=\;\omega_{s,G^{\prime }ite}\right)}$$

We used atomic force microscopy (AFM) to analyze the thickness of the graphene nanosheets after stabilization with BSA. The height-profile images of the graphene sheets revealed that they had a thickness of 7.6 ± 2.3 nm, suggesting the presence of few-layer graphene sheets in aqueous dispersion (Figure 2F). Note that the relatively high thickness of fabricated graphene is due to the absorption of BSA on its surface [38].

### 3.2. Scale-up Study

**Figure 3:**
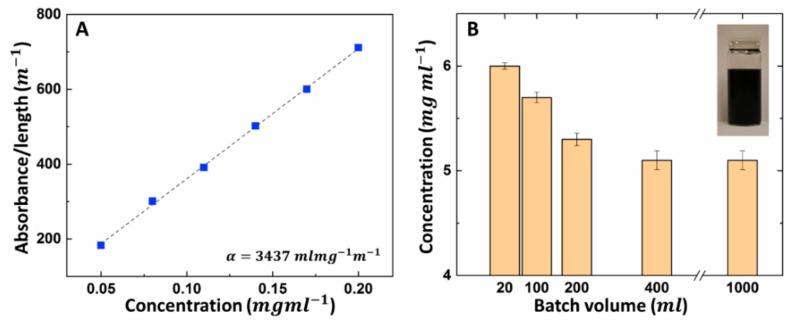
Graphene concentration measured given by ultraviolet (UV) absorption. (**A**) Absorption coefficient obtained from ultraviolet-visible (UV-vis) spectroscopy at *λ* = 660 nm. (**B**) Change of graphene concentration with respect to batch sizes varying from 20 mL to 1000 mL. The resultant concentration of wet ball-milled graphene remains constant (5.1 mg mL) for batches larger than 400 mL.

A significant factor in the process of exfoliating graphene from pristine graphite is the scalability of the production method. The bio-graphene presented here preserves its qualities, such as consistent yield, lateral size, conductive characteristics and being devoid of defect. In order to increase the batch volume, the number of balls was chosen based on the ratio of the overall surface area of the balls to the volume of the solvent ($r_{bs}$). Graphene ink was produced in batch volumes varying from 20–1000 mL with $r_{bs}\;=\;500\;\pm \;01\;{\mathrm{mm}}^{2}/{\mathrm{mm}}^{3}$. Measured conductivities remained unchanged and the printed graphene remained stable under flexing. Figure 3 demonstrates graphene yield from the wet ball milling taken from various volumes, as measured via UV-vis spectroscopy. It appears that more graphite particles left the mixture as sediment in larger batches. However, the production method can lead to a graphene concentration of $5.1\;{\mathrm{mg}\;\mathrm{mL}}^{-1}$ regardless of volume for batches greater than 200 mL. Concurrently, the change of lateral size in different batch sizes can contribute to the quality and scalability of the production method. Hence, Raman spectra of the samples in different batch volumes were used to statistically estimate the mean lateral size of the graphene platelets (Figure 2D and Figure S2). This lateral size (approximation further confirms the TEM findings (Figure 2B and Figure S3), as liquid phase exfoliation typically results in a distribution of flake sizes in each sample.

### 3.3. Ink Formulation and Printing

**Figure 4:**
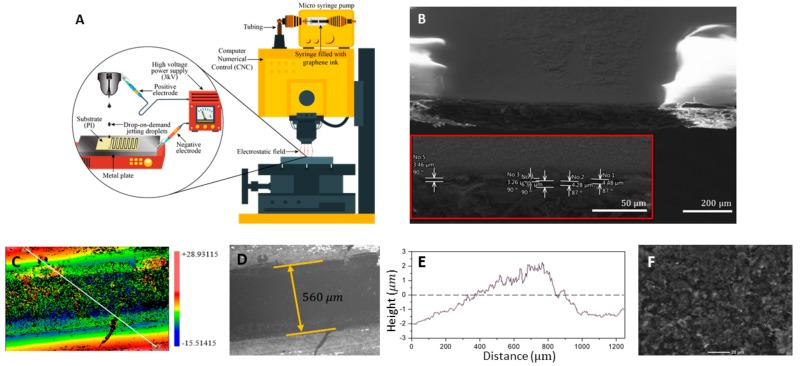
(**A**) Drop-on-demand printer setup for the inkjet printing of graphene (**B**) SEM for cross-sectional view of printed patterns with magnifications which show a normal height of ≈4 μm (**C**) Height contour of full printed graphene and sample line through which an average height is reported. (**D**) The 20× magnified section of the sample print. (**E**) In distance 0 and 1200 on the sample line (c), the height is measured over the substrate. The maximum measured height is the peak of the printed material. The difference between these two indicates the height of the print (≈4 μm) at maximum which is compatible with SEM imaging. (**F**) SEM for THE top view of printed graphene after annealing at 280 °C for 30 min; scale bar: 20 μm.

**Figure 5:**
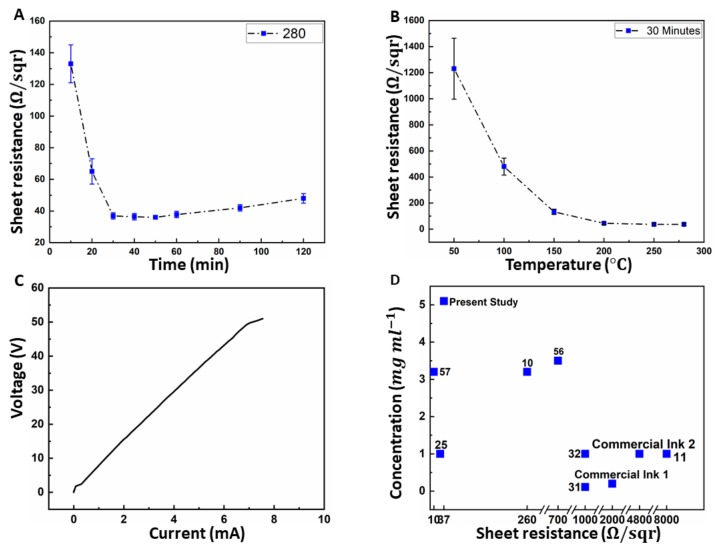
(**A**) Change of sheet resistance $R_{S}$ of printed graphene with respect to annealing time in 280 °C oven. (**B**) Change of the sheet resistance of printed graphene with respect to changes in annealing temperature. (**C**) Linear I–V response of the lines with length of 100 mm measured by a potentiostat. (**D**) Comparison of the yield and resultant sheet resistance of produced graphene with other published approaches [10,11,25,31,32,55,56,57]. In the studies that sheet resistance was not reported directly, their values were estimated by Equation (7) using the reported resistivity/conductivity values.

Graphene ink was applied to Kapton PolyIimide (PI) using a custom designed printing setup demonstrated schematically in Figure 4. As the produced FLG aqueous solution is negatively charged, surface modifications to PI were crucial for improving the hydrophilicity of the substrate prior to printing [14]. Traditionally, conductive inks are printed using the commercial ink out of the nozzle onto the substrate either mechanically or thermally [36,47]. It is commonly reported that microscale cracks appear within the printed lines, rendering them susceptible to the deflection of the substrate. In many studies, polymer stabilizers such as ethyl cellulose are added to the ink with the purpose of crystallization at the time of annealing [14,47]. Such binders are often insulators, and may not be the most effective post-processing method when it comes to the electronic applications of graphene [10,54]. In order to address the print instability issue and avoid numerous printing passes, we have utilized an electrostatic field to fix the conductive ink on the substrate producing graphene lines on bendable substrates without binders (Figure 1B). In one pass, graphene ink was lain onto the substrate and was immediately fixed after, via thermal annealing, in a preheated oven at the temperatures and durations given in Figure 5A. In order to consolidate graphene platelets on the substrate, a potential difference with a magnitude of 3 kV was created between the needle and the aluminum film placed under the substrate. These served as positive and negative electrodes, respectively. 

The chosen PI substrate was 0.06 mm thick. The tip of the flat-ended needle was set to 1.2 ± 0.1 mm from the top surface of the metal plate, and its perpendicularity was ensured by the CNC machine. The induced electrostatic force causes liquid meniscus at the interface to form a micro-droplet, which combines with the acceleration due to gravity to overcome the surface tension of the liquid, and is pushed towards the substrate [44]. Due to the surface tension of the graphene ink, the positive pressure, caused by the syringe pump, cannot result in a constant flow of the ink (9 μL/s in the reported experiments). In the absence of an electrostatic field, large millimeter-sized drops leave the tip of the needle only after the accumulation of ink after several seconds. Thus, the flow of the ink can be controlled by the presence of the potential difference between the electrodes. The advantage of the printing setup used in this study, as compared to common commercial inkjet printers, can be observed in the SEM results. Figure 4F shows minimal disorientation of graphene platelets in post-baked printed patterns, which is advantageous in reducing micro-scale junctions.

The resolution of prints is controlled by: the inner diameter of the chosen needle, the positive pressure behind the ink-filled syringe (represented by the flow rate injected by the syringe pump), needle speed along the printed lines and the electric potential difference between the needle’s tip and the substrate (see the Supplementary Materials for more details). The width of the prints was measured to be 550 ± 50 μm in all of the cases presented here.

### 3.4. Post Processing and Electrical Conductivity

The normal height of printed samples directly influences the overall conductivity of graphene lines and their quality. A thick print might collapse and crack after bending, and the coffee ring effect will cause an unwanted inconsistency in the thickness of the side edges of the prints [25,32]. The Profilometry results (Figure 4C–E and Figure S5) show that the maximum thickness occurs in the center line of the prints, which indicates that the majority of graphene flakes are deposited in the middle, thereby proving that there was no observable coffee ring effect. This is a stable cross section under tangential stresses across the surface of substrates during flexing.

Post processing is essential for the improvement of the conductivity the and stability of the printed FLG. On one hand, it leads to the release of possible oxide compounds; and on the other hand, results in the BSA burn-off, which is essential for maintaining the stability of the patterns when in touch with water. During thermal decomposition, all amino acids emit volatile gases, mainly $H_{2}O$ and ${\mathrm{NH}}_{3}$. Nevertheless, the mass of the residues is heavily dependent upon the temperature to which they were exposed. At 280 °C, the residue of some amino acids (i.e., Cysteine) can be reduced to 10% of their initial mass [58]. Thermal annealing reduces flake-to-flake and flake-to-substrate defects while improving the order of the surface morphology [10,22,59]. The prints made from FLG ink were once annealed in various durations ranging from 10 to 120 min in a constant temperature of 280 °C (Figure 5A), and once annealed for 30 min in temperatures varying from 50 °C to 280 °C (Figure 5B). As a thermally stable organic substrate, PI is popular among the developers of flexible printed circuits due to its capability to maintain its stability across a wide range of temperatures (from –269 °C to +400 °C) [55]. Therefore, it is safe to assume that the quality of the substrate is not compromised during the annealing process. 

Graphene is naturally conductive, due to the fact that the connection of each carbon atom forms three μ-bonds, leaving one out-of-plane electron (π bond) free in the valence shell. The end to end resistance of the lines were measured via a potentiostat (Figure 5C) for lines with mean dimensions of 100 × 0.56 × 0.004 mm. Equation (7) can be used to give the conductivity of the processed graphene σ ≈ 6800 S/m, for which, l is the length of the lines, w and t are the width and height of the lines respectively, and R is the measured resistance. Both w and t were measured by the profilometer (Figure 4D) at five distinct points along the printed lines, and the mean values were used for the conductivity measurement. The results show that, although depositing layers of wet ball-milled graphene on substrate has a sheet resistance of 133 Ω/sqr, annealing the samples in a standard oven up to 280 °C  can reduce the sheet resistance to as low as 36.75 Ω/sqr.

(7) $$\sigma \;=\;\frac{l}{wtR}$$

It should be noted that, without surface preservants, the FLG prints reported in this study could be detached from the substrate if scratched or repeatedly folded. We observed that the printed lines could retain their conductivity under flexing (Figure 1B) and remained stable after being submerged under DI water with PH = 7 for over 7 days as a result of BSA burn-off at high temperature (Figure 1E). While there are already commercial graphene inks available, they possess lower concentrations, as well as dramatically higher sheet resistance. The concentration and sheet resistance for commercial ink1 and ink2 (Supplementary Materials) are $C\;=\;0.2\;{\mathrm{mg}\;\mathrm{mL}}^{-1}$ and $R_{s}=2,\;4.8\;k\Omega /\mathrm{sqr}$ respectively (both after thermal processing). Figure 5D [11,25,31,32,55,56,57], demonstrates a comparison of the production method in this study with other published records, both in terms of concentration (mg/mL) and the resultant sheet resistance.

## 4. Conclusions

The production of defect-free and stable graphene dispersion in aqueous medium is vastly desired for biological applications. Particularly, applying conductive graphene patterns on flexible substrates can aid the electrophysiological study on neuronal cells by resolving the reported mechanical mismatch between biosensors and the soft cell membrane. Here we demonstrated a facile graphene production method by combining BSA with wet ball milling for the first time, and further inkjet printed the prepared ink on a flexible polymer, PI. The exfoliation process starts from graphite particles with the concentration of 20 mg/mL, and results in 5.1 mg/mL of stable graphene dispersion. The as-prepared graphene sheets possess a lateral size on the order of hundreds of nm and are 2–3 layers on average. The produced dispersion remained stable for weeks, which is advantageous during the printing process, as it dramatically lessens the likelihood of clogging issues.

The utilized custom-designed inkjet printer benefits from an electrostatic force to consolidate the conductive patterns on the substrate. Moreover, the effect of thermal annealing was explored in different durations and temperatures to reduce the sheet resistance of graphene patterns to 36.75 Ω/sqr, while enhancing their binding to the substrate. The resultant conductive lines did not lose their conductivity, nor adhesion to PI when in touch with water, which is desirable as cell media are typically water-based.

In a recent study, our group observed that rat dopaminergic (N27) neuronal cells live compatibly and adhere to the graphene patterns (Figure S4) in the biosensing platforms that are made based on the aforementioned methodology [45]. These cells are commonly used in vitro, as models for Parkinson’s disease. Thus, the reported methodology can set the ground for further investigations about electrical signals for cell communications and other applications regarding flexible/printable electronics.

## Supplementary Materials

The following are available online at https://www.mdpi.com/2079-6374/10/1/6/s1, Figure S1: The ID/ID’ ratio in different batch sizes, Figure S2: Lateral size of graphene platelets produced by wet ball milling, Figure S3: TEM imaging of shear exfoliated graphene, Figure S4: Live–dead cell assay, Figure S5: Cross-section of graphene lines captured by SEM.
