# Supplementary material for: High-Yield Production of Aqueous Graphene for Electrohydrodynamic Drop-on-Demand Printing of Biocompatible Conductive Patterns
Source: Biosensors (Basel). 2020 Jan 17;10(1):6. doi: 10.3390/bios10010006 (PMC7167870; doi:10.3390/bios10010006)
Supplement: Supplementary file 1 [file biosensors-10-00006-s001.pdf]

Supplementary information

High-Yield Production of Aqueous Graphene for Electrohydrodyamic Drop-on-Demand Printing of Biocompatible Conductive Patterns

Amir Ehsan Niaraki Asli[1], Jingshuai Guo[1], Pei Lun Lai[1], Reza Montazami[1], Nicole N. Hashemi[1,2,*]

1 Department of Mechanical Engineering, Iowa State University, Ames, IA 50011, USA; niaraki@iastate.edu (A.E.N.A); jguo1@iastate.edu (J.G.); issaclun@iastate.edu (P.L.L.); reza@iastate.edu (R.M.); nastaran@iastate.edu (N.N.H.)

2 Department of Biomedical Sciences, Iowa State University, Ames, IA 50011, USA;

* Correspondence: nastaran@iastate.edu

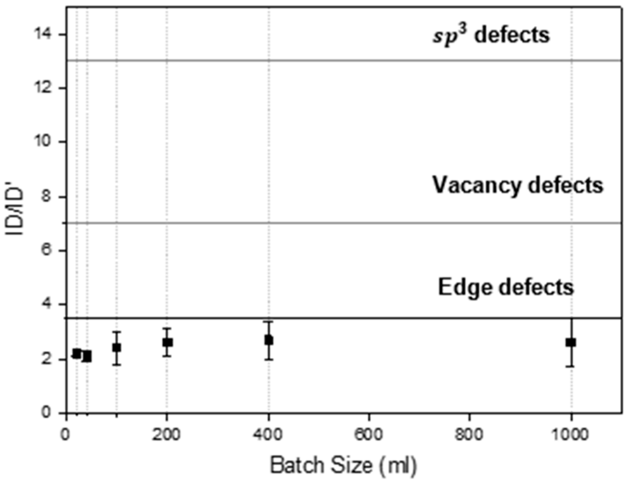

Figure S1. The ID/ID' ratio in batch sizes of 20, 40, 80, 200, 400, 1000 ml. The samples were taken on 5 different trials.

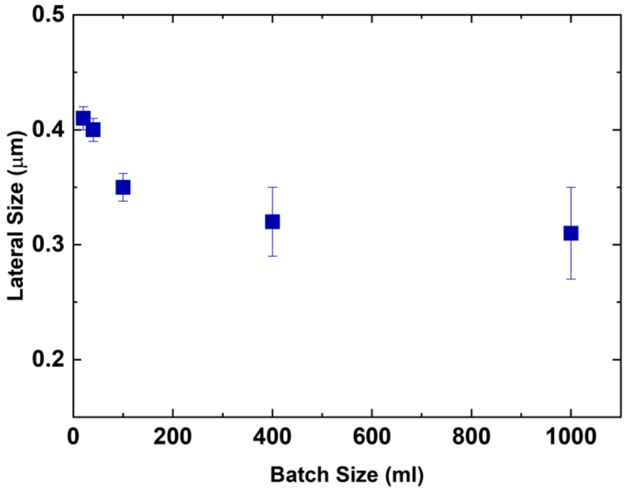

Figure S2. The lateral size of graphene platelets produced by wet ball milling was calculated to be $\langle L \rangle > 0.3\ \mu m$ regardless of batch size. Subsequent to determining defect source in shear exfoliated graphene, Raman spectra can statistically be analyzed to give mean lateral size of graphene platelets ($\langle L \rangle$).

The lateral size of graphene platelets were calculated using Equation 1.[46] While, k is the experimentally measured value set equal to 0.17 and $(I_D/I_G)_{Graphite}$ has been measured equal to 0.02 from the Raman spectra of raw graphite powder.

$$\langle L \rangle = \frac{k}{\left(\frac{I_D}{I_G}\right)_{Graphene} - \left(\frac{I_D}{I_G}\right)_{Graphite}} \quad (1)$$

Please do not adjust margins

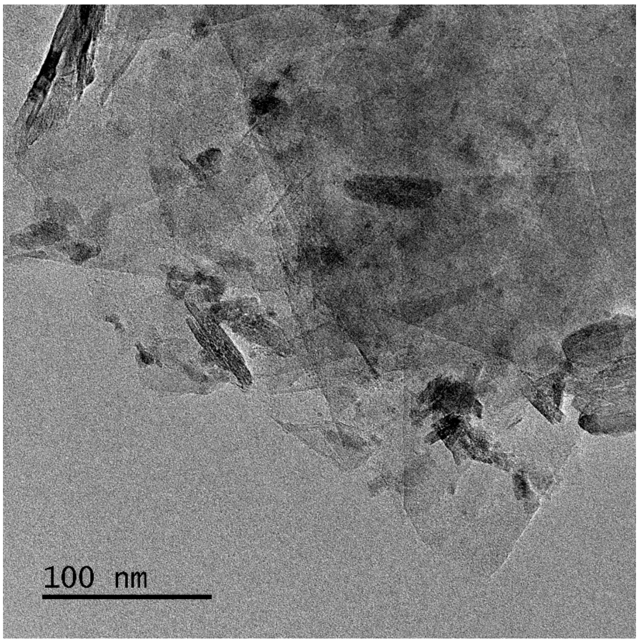

Figure S3. TEM imaging of shear exfoliated graphene demonstrates a distribution of graphene flake sizes. While, thinner and laterally larger flakes are more desirable, the graphene sheets tend to stack up after exfoliation; Scale bar: 200 nm.

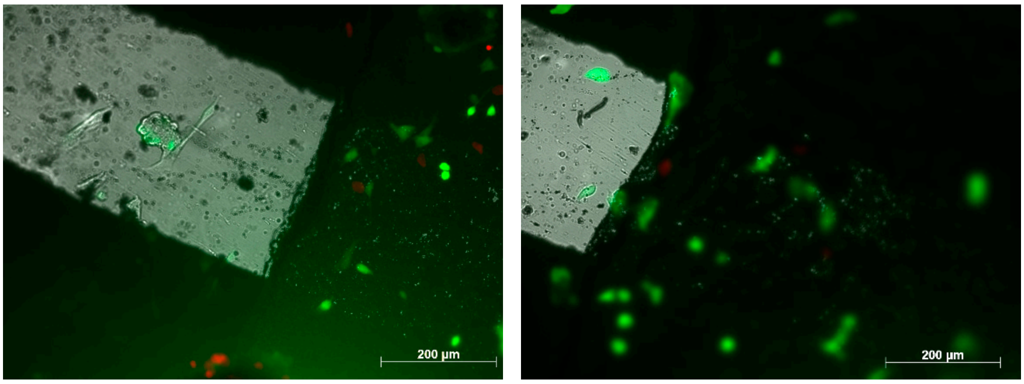

Figure S4. Live–dead cell assays were performed using a 70 µM CellTracker™ CMFDA solution combined with an 8 µM propidium iodide (PI) solution in FBS-free RPMI medium. N27 cells in vitro after $72h$ at 37 $°C$ in a 5% CO2 environment deposited on graphene prints. Live cells are shown in green, and dead cells are in red; scale-bars: 200 μm. For further details on cell imaging and deposition process refer to reference 45.

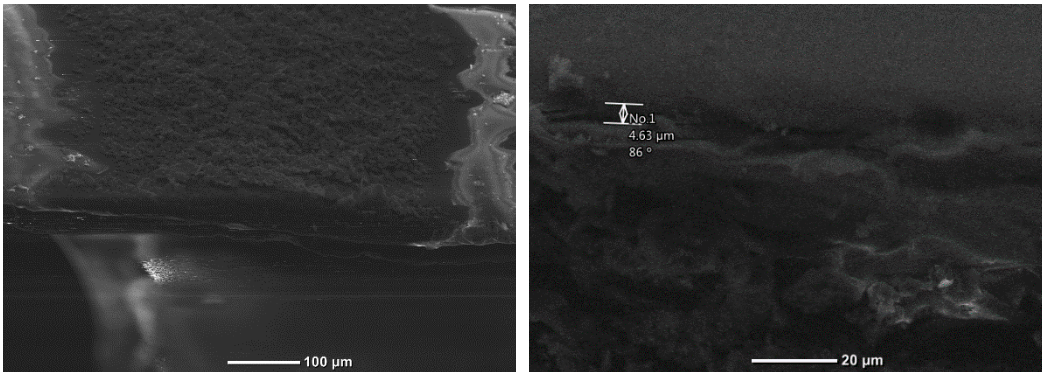

Figure S5. Cross-section of graphene lines captured by SEM. Homogeneous normal height of graphene along the line is demonstrated. The majority of the graphene ink is deposited along the centreline, which resulted in dome-like profile. Left image scale bar: 100 μm; Right image scale bar: 20 μm.

This journal is © The Royal Society of Chemistry 20xx

Please do not adjust margins
